# Evaluating the effects of PeakATP^®^ supplementation on visuomotor reaction time and cognitive function following high-intensity sprint exercise

**DOI:** 10.3389/fnut.2023.1237678

**Published:** 2023-08-04

**Authors:** Jessica M. Moon, Trevor J. Dufner, Adam J. Wells

**Affiliations:** Exercise Physiology, Intervention, and Collaboration Lab, School of Kinesiology and Rehabilitation Sciences, University of Central Florida, Orlando, FL, United States

**Keywords:** multiple object tracking, mood, performance, ATP, cognition

## Abstract

The purpose of this study was to examine the effects of 14-days adenosine 5′-triphosphate (ATP) supplementation (PeakATP^®^) on reaction time (RT), multiple object tracking speed (MOT), mood and cognition. Twenty adults (22.3 ± 4.4 yrs., 169.9 ± 9.5 cm, 78.7 ± 14.6 kg) completed two experimental trials in a double-blind, counter-balanced, crossover design. Subjects were randomized to either PeakATP^®^ (400 mg) or placebo (PLA) and supplemented for 14-days prior to each trial. During each trial, subjects completed a three-minute all-out test on a cycle ergometer (3MT), with measures of visuomotor RT [Dynavision D2 Proactive (Mode A) and Reactive (Mode B) tasks], MOT (Neurotracker), mood (Profile of Mood States Questionnaire; POMS) and cognition (Automated Neuropsychological Assessment Metrics; ANAM) occurring before (PRE), immediately post (IP) and 60 min post-3MT (60P). Subjects ingested an acute dose of the assigned supplement 30 min prior to completing PRE assessments for each trial. Trials were separated by a 14-day washout period. PeakATP^®^ significantly attenuated declines in hits (*p* = 0.006, η_p_^2^ = 0.235) and average RT (AvgRT, *p* = 0.006, η_p_^2^ = 0.236) in Mode A, significantly improved AvgRT (*p* = 0.039, η_p_^2^ = 0.174) in Mode B, and significantly reduced the total number of misses (*p* = 0.005, η_p_^2^ = 0.343) in Mode B. No differences between treatments were noted for MOT, POMS or ANAM variables. In conclusion, these results indicate that PeakATP^®^ maintains proactive RT and improves reactive RT following high-intensity sprint exercise suggesting that supplemental ATP may mitigate exercise induced cognitive dysfunction.

## Introduction

Successful performance in sport is dependent upon the integration of athletic ability and several cognitive processes including decision-making, perception, anticipation, attention, and working memory (1). Action anticipation, defined as the ability to observe and predict the behaviour of other individuals or objects (2), is particularly important as it integrates several cognitive processes, which subsequently coordinate and dictate the execution of an ensuing motor action. In most sports, athletes are exposed to cognitively taxing conditions resulting from the combination of physical, environmental and psychological demands. In particular, high intensity sprint exercise (HISE) has been shown to elicit transient cognitive dysfunction, manifesting as fatigue-associated lapses in concentration, decision making, and skill performance (3–5). Considering that repeated bouts of HISE are fundamental to many sports, the ability to capture and anticipate changes in cognitive function during and following HISE are necessary. Likewise, characterizing the duration of cognitive impairment resulting from HISE is necessary, as changes in cognition may manifest early during athletic events following HISE efforts. Understanding the immediate and transient effects (i.e., within a period of time typical to competitive games/matches) is important for the adequate allocation of rest for cognitive recovery, as well as the institution of game and player management. This information also has implications for multi-heat events and single day multi-game events. In tandem, the ability to accurately and reliably assess changes in cognitive performance is critical for the effective examination of interventions designed to mitigate cognitive dysfunction and maintain sports performance.

Given the dynamic nature of the sporting environment and variability in the physical and psychological demands within and between competitive events, repeatable laboratory-based exercise protocols are often utilized alongside cognitive tasks to assess subsequent perturbations in cognitive performance. For example, Mekari et al. (6) reported that nine minutes of cycling at 85% of peak power output (PPO) resulted in significant impairments in reaction time during a modified Stroop task when compared to low (40% PPO) or moderate (60% PPO) intensity cycling. Similarly, Sun et al. (7) reported that HISE consisting of a 10 by 6 s repeated Wingate protocol with 30 s of passive recovery between bouts significantly decreased response accuracy on a Go/No-Go task. Further, in a series of experiments by Dietrich and Sparling (8), it was reported that exercising at 70%–80% of maximum heart rate (MHR) for 45 min resulted in significantly more errors on the Wisconsin Card Sorting Task during exercise among cyclists and runners, and significant deficits in working memory and attentional focus during a Paced Auditory Serial Addition Task when compared to non-exercising controls. Collectively these investigations suggest that regardless of the exercise stimulus, if the intensity is sufficient, transient dysfunction of several cognitive processes, including reaction time, sustained attention, working memory, response accuracy and response inhibition may manifest. Given that these processes are integral for sport specific task performance, interventions directed at mitigating deficits in these cognitive domains during sport are warranted. Moreover, in consideration of the need to precisely match the physiological demands of activity across repeated trials, lab-based exercise protocols may be preferred.

In the brain, cognitive processes are tightly coupled to ATP metabolism where energy fluctuations are correlated with changes in energy demand (9). Therefore, the brain may be particularly vulnerable to disturbances in the availability of energy resources. Accordingly, interventions that have the potential to sustain ATP metabolism may be useful for attenuating cognitive dysfunction resulting from HISE. Oral disodium ATP supplementation may be a potentially novel nutritional intervention against transient HISE induced cognitive dysfunction, having previously shown ergogenic potential for cardiovascular health (10), muscular performance (10–12), and the attenuation of fatigue (13) when 400 mg is consumed daily. Chronic oral ATP supplementation, 30 days with 5 mg/kg/day, has been shown to increase portal vein ATP concentrations and nucleoside uptake by red blood cells in animal models, resulting in an increase in ATP synthesis within these cells, despite no elevation in plasma ATP concentrations (14). Further, vessel dilation and enhanced post-exercise blood flow have been reported to increase following supplementation with disodium ATP in humans (15) with many speculating that the ergogenic effects observed in previous investigations stem from this hemodynamic change (11, 13). With changes in regional cerebral blood flow and oxygenation purported as potential mechanistic elements underpinning acute high intensity exercise associated cognitive dysfunction (16, 17), along with the reported hemodynamic effects of supplemental disodium ATP and the potential for enhanced ATP delivery during HISE, an examination of the effects of supplemental disodium ATP on measures of cognition following HISE is warranted. The purpose of this study, therefore, was to examine the acute effects of disodium ATP (400 mg PeakATP^®^) on reaction time, multiple object tracking, mood, and cognitive function, in healthy men and women following an acute bout of HISE. We hypothesized that PeakATP^®^ would attenuate post-exercise declines in reaction time, mood disturbances, multiple object tracking speed, and cognitive function when compared to placebo.

## Materials and methods

### Experimental approach to the problem

**Figure 1:**
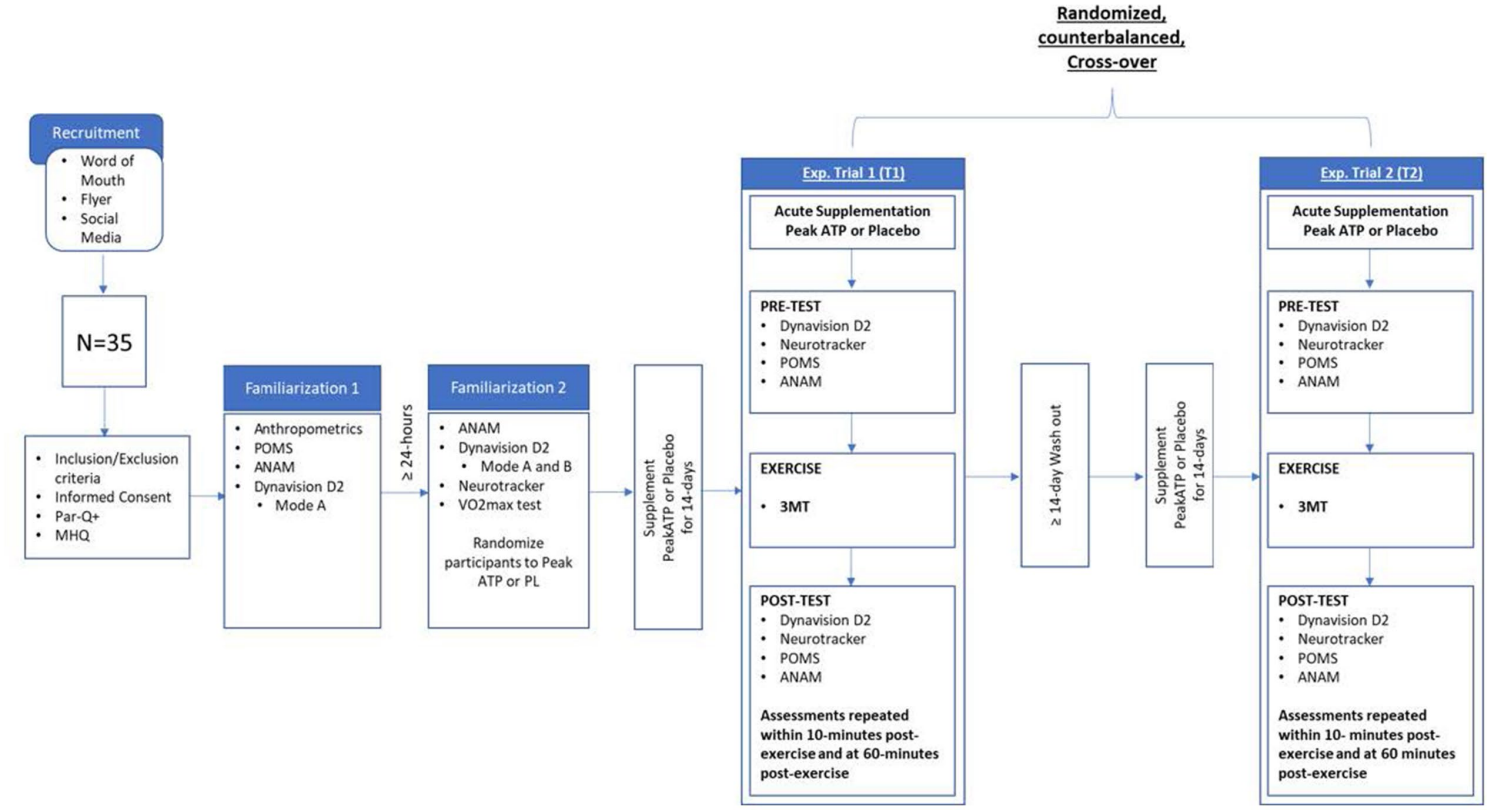
Research design overview. MHQ, medical health questionnaire; 3MT, 3 min all out test; POMS, profile of mood states; ANAM, automated neuropsychological assessment metrics; Exp, experimental.

This study followed a randomized, double-blind, cross-over design. Subjects completed a total of five visits to the study site. The initial visit (visit 1) consisted of informed consent and eligibly screening. Eligible subjects were instructed to abstain from caffeine for 24-h and asked to be two hours fasted prior to all subsequent visits (visits 2–5). During visit 2, subjects underwent anthropometric assessments of height, weight, and body composition and were familiarized with the Profile of Mood States questionnaire (POMS), the Automated Neuropsychological Assessment Metrics (ANAM) assessments, and the Dynavision D2 Mode A reaction time assessment. At least 24 h later, subjects returned for visit 3, during which they underwent additional familiarization with the ANAM and Dynavision D2 Mode A assessment, as well as initial familiarization with the Dynavision D2 Mode B reaction time assessment and Neurotracker multiple object tracking (MOT) assessments. A maximal aerobic power assessment was then performed. Following visit 3, subjects were randomly assigned by sex in a counterbalanced fashion to either PeakATP^®^ or placebo (PLA) and were instructed to consume their assigned supplement for a period of 14-days. Following supplementation, subjects reported back to the laboratory within 24 h of their last dose to complete the first of two experimental trials (visits 4 and 5). During each experimental trial, they also consumed an acute dose of their assigned supplement 30 min before completing pre-exercise (PRE) assessments on the Dynavision D2 (Mode A & Mode B), MOT, POMS, and the ANAM. Immediately following PRE assessments, subjects completed a standardized warm-up followed by a three-minute all-out high-intensity cycle test (3MT). Subjects then repeated PRE assessments immediately-post (IP) and 60-min post exercise (60P). Upon completion of the first experimental trial, subjects underwent a two-week washout period, followed by 14 days of supplementation with the alternate supplement (PeakATP^®^ or Placebo). Subjects returned to the study site within 24 h of their last dose to complete experimental trial 2 (visit 5). Experimental Trial 2 was administered in an identical fashion, at a similar time of day, to Experimental Trial 1. This study was registered with clinicaltrials.gov under the identifier NCT05100589. A schematic of the experimental study protocol is depicted in Figure 1.

### Subjects

**Figure 2:**
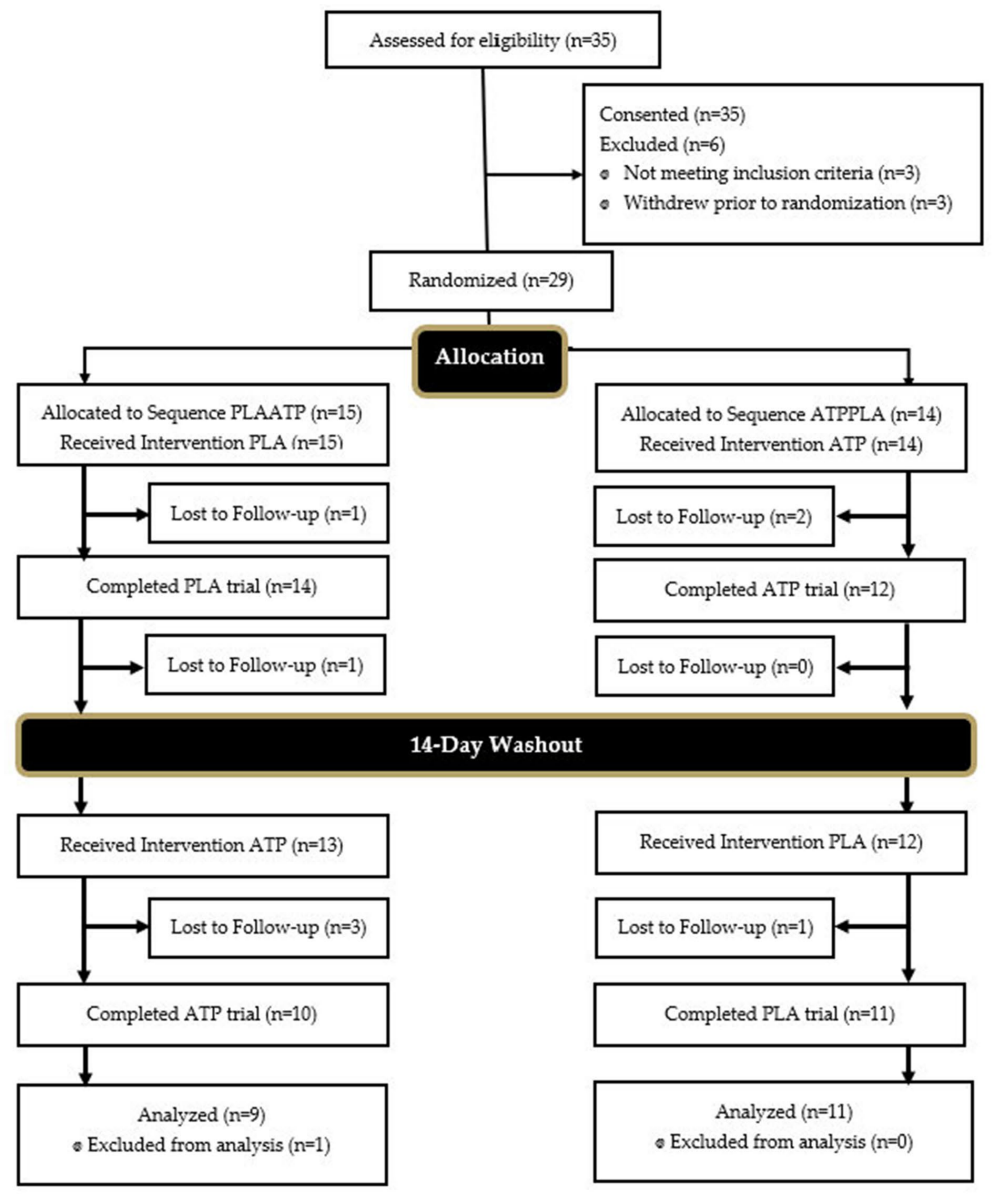
Consolidation standards of reporting trials (CONSORT) diagram. PLA, placebo; ATP, PeakATP; PLAATP, participant first received PLA followed by ATP; ATPPLA, participant first received ATP followed by PLA.

A Consolidation Standards of Reporting trials (CONSORT) diagram for all study recruitment, randomization and project completion is provided in Figure 2. A total of 20 healthy, recreationally active subjects, (ten males, ten females) between the ages of 18 and 40 (22.3 ± 4.4 yrs., 169.9 ± 9.5 cm, 78.7 ± 14.6 kg, 27.0 ± 9.5% fat) successfully completed the study protocol. Preceding participation, subjects provided informed consent using an approved IRB consent document (Study # 3272; Approval Date: 10.6.2021). Subjects were allowed to undergo participation in study protocols if they were free from all pulmonary, cardiovascular, autoimmune, musculoskeletal, gastrointestinal, or other diseases or disorders, as reported in their medical history questionnaire (MHQ). Additionally, subjects had to report performing at least 150 min of exercise each week meeting the American College of Sports Medicine standard for recreationally active individuals and be determined as ready for physical activity through completion of the PAR-Q assessment. Subjects had to abstain from dietary supplement use including creatine and beta-alanine for a minimum of 4 weeks prior to beginning research protocols.

To achieve a statistical power (1 −β) of 0.95, a total sample size of 17 subjects was required for this study. Power calculations were performed *a priori* using G*Power software (3.1.9.4, HHU, Dusseldorf, Germany) and were based on an effect size (dz) of 0.963 derived from changes in Go/No Go accuracy from pre- to post-acute high-intensity exercise under normoxic condition reported by Sun et al. (7).

### Procedures

#### Anthropometrics

Height and weight were assessed during visit 2 using a stadiometer and scale (Health-o-meter Professional Patient Weighing Scale, Model 500 KL, Pelstar, Alsip, IL, United States) while body composition was assessed via bioelectrical impedance analysis (BIA; InBody 770, Biospace Co, Ltd. Seoul, Korea). Subjects were asked to be 2 h fasted and well-hydrated. Prior to testing subjects voided their bladder, and removed shoes, socks, and jewelry.

#### Maximal aerobic power testing

During visit 3, subjects performed a ramp protocol to volitional fatigue on a cycle ergometer (Lode, Excalibur Sport, Groningen, Netherlands). Prior to testing, subjects completed a warm-up consisting of 5 min of light cycling at an intensity of 50 watts (W) at a self-selected pace; 10 body weight squats, 10 body weight walking lunges, 10 dynamic waking hamstring stretches, and 10 dynamic walking quadricep stretches. The MAP protocol required subjects to maintain a pedaling cadence of 70–80 revolutions per minute (RPM) at an initial workload of 100 W. The workload was increased 30 W per minute (1 W/2 s) until the subject was unable to maintain a cadence above 70 RPM for 10 s despite verbal encouragement or volitional fatigue. Expired gasses were analyzed using open-circuit spirometry (True One 2,400^®^ Metabolic Measurement System, Parvo-Medics Inc., Sandy, UT) Subjects were connected to the metabolic cart via a measured and fitted facemask covering both the nose and mouth (V2 Mask^™^, Hans Rudolph, INC, United States), and a breathing tube. Peak power output (PPO) was recorded as the highest power output achieved in W. Gas exchange threshold (GET) was determined via computerized regression analysis of the slopes of CO_2_ uptake (VCO_2_) vs O_2_ (VO_2_) uptake. Power at the GET was recorded. Seat height was recorded to standardize cycle dimensions for subsequent cycle assessments.

#### Three-minute all-out cycle assessment (3MT)

During each experimental trial (visits 4 and 5), subjects completed a 3MT (Lode, Excalibur Sport, Groningen, Netherlands) (18). Subjects completed a standardized warm up identical to that performed during the MAP assessment. The assessment began with an initial preparation phase consisting of 60 s of cycling (50 W, 70–80 rpm). During the last 5 s of the preparation phase, participants were instructed to pedal as maximally as possible before immediately completing the 3MT. Resistance during the 3MT was set as a function of pedaling rate using a scaling factor based on the power output at a set cadence of 80 RPM being equal to 50% of the difference between the power output at GET and PPO assessed during the MAP test (Eq 1). Strong verbal encouragement was provided through the duration of the assessment, while the subjects were blinded to the elapsed time of the assessment to prevent pacing. Upon completion of the 3MT, subjects remained on the cycle ergometer and completed 3 min of unloaded cycling at a self-selected pace.


(1) $$ResistanceFactor=\frac{\left(\left(PPO-PPO_{GET}\right)*0.50\right)+PPO_{GET}\;}{80^{2}}$$

#### Dynavision D2 assessments

Reaction time was assessed using the Dynavision D2 visuomotor training device. The Dynavision D2 consists of a 4 ft. × 4 ft. computer integrated board with 64 tactile light emitting targets arranged into five concentric rings. During a test, illuminated targets serve as a visual stimulus that require a physical hand strike to extinguish.

##### Mode A

The Mode A proactive reaction time task required subjects to recognize and respond as fast as possible to random and sequential appearing stimuli across the Dynavision apparatus target field. Following a five-second visual countdown on the boards t-scope, an initial stimulus presented on the D2 board in a random location. The stimulus remained illuminated until the button was struck by the participant, following which the stimulus then appeared in another random location. The participant was instructed to successfully identify and strike as many stimuli as possible within 60 s with both hands.

##### Mode B

Similar to Mode A, the Mode B reactive reaction time task required subjects to respond as fast as possible to random and sequential stimuli across the target field. However, during the Mode B assessment, the stimulus remained lit for only one-second before changing position to another random location within the target field. In addition, the Mode B test included a cognitive stressor in the form of a five-digit number that subjects were required to recite during the test. The five-digit number was presented on the center of the screen (T-scope) of the D2 apparatus 11 times during each 60 s trial and remained on the screen for 0.75 s.

To eliminate learning and training effects, subjects completed ten Mode A assessments during familiarization visit one, and a further eight Mode A assessments during familiarization visit two (19). Additionally, three Mode B assessments were completed during familiarization two. Mode A and Mode B assessments were completed at PRE, IP, and 60P during both experimental trials. For Mode A, the number of hits and average reaction time per hit were assessed. For Mode B, the number of hits, misses, and average reaction time per hit, were assessed. The average of three discrete tests was utilized for each assessment at each time point.

#### Multiple object tracking

MOT speed was assessed via the completion of a CORE assessment using Neurotracker MOT software (CogniSens Athletic, Inc., Montreal, Quebec, Canada). The Neurotracker MOT assessment required subjects to track four targets among eight spheres projected within a cube space, subtending a visual angle of 30°. The spheres followed a linear trajectory in space, with deviations in trajectory occurring in response to collisions between balls and with the walls of the cube. A fixation spot was presented in the center of the cube to support an effective distribution of attention throughout each trial. The CORE assessment included 20 discrete trials and lasted approximately six min. During each trial, speed of displacement increased or decreased in a staircase fashion depending on whether the participant correctly identified all indexed targets. After each correct response, the speed of ball displacement increased by 0.05 log units and decreased by the same proportion following incorrect responses. The MOT speed threshold, defined as the speed at which the participant correctly identified all target balls 50% of the time (i.e., the point at which their performance neither improves nor deteriorates) was determined. Each trial was performed in a private room, free of distractions. Baseline performance, calculated as the geometric mean of three back-to-back CORE assessments (60 trials total), was established during visit 3. CORE assessments were also completed at PRE, IP, and 60P during both experimental trials (visits 4 and 5).

#### Profile of mood states questionnaire

Mood state was assessed through the administration of the Profile of Mood State (POMS) questionnaire. The POMS consists of 58 words or phrases in a Likert format soliciting responses regarding how the participant feels at the time of completion (0–4, 0 = Not at all, 1 = A little, 2 = Moderately, 3 = Quite a bit, 4 = extremely) and provides measures of tension, depression, anger, vigor, fatigue, and confusion. Total mood disturbance (TMD) was calculated by subtracting vigor from the sum of the 5 other negative mood states and adding 100 to avoid a negative result. Subjects completed a POMS questionnaire during visit 2 as well as at PRE, IP, and 60P during both experimental trials (visits 4 and 5).

#### Automated neuropsychological assessment metrics assessments

Cognitive performance was assessed via ANAM software (ANAM v.4.0; Vista Life Sciences, Parker, CO). The ANAM core battery was utilized for this study, which consisted of a concussion symptoms index (CSI) and eight cognitive subtests. The list of subtests, abbreviations and measures/cognitive domains associated with the ANAM core battery are presented in Table 1. The CSI was utilized to assess the degree of psychological stress and consisted of 12 symptoms scored on a 7-point Likert-type scale from 0 (absent) to 6 (severe). Symptoms included headache, nausea, balance problems/dizziness, fatigue, drowsiness, feeling like “in a fog”, difficulty concentrating, difficulty remembering, sensitivity to light and noise, blurred vision, and feeling slowed down. Previous literature has indicated that post concussion-like symptoms are not unique to mild head injury, with an increase in self-reported symptoms having been reported in healthy individuals who are experiencing high levels of perceived stress or mental fatigue (20, 21).

**Table 1:** ANAM^™^ battery subtests and associated measures/cognitive domains.

Subtest	Abbreviation	Measure/cognitive domain
Concussion symptom inventory—percentage of endorsed symptoms	CSI_%_	Psychological stress
Concussion symptom inventory—sum of severity ratings	CSI_SUM_	Psychological stress
Simple Reaction Time	SRT	Processing speed
Code Substitution-Learning	CSL	Visual search, sustained attention, working memory, processing speed
Mathematical Processing	MATH	Computational skills, concentration, working memory
Code Substitution-Delayed	CSD	Sustained attention, working memory, short term memory and learning
Procedural Reaction Time	PRT	processing speed
Matching to Sample	M2S	Spatial processing, visuospatial working memory
Simple Reaction Time-Repeat	SRT2	Cognitive fatigue, processing speed
Go-No-Go	GNG	Response inhibition

ANAM^™^, automated neuropsychological assessment metric.

Subjects completed the ANAM test battery on two separate occasions (visits 2 and 3) to establish reliable testing and baseline cognitive scores, which is consistent with previous literature (22, 23). ANAM cognitive tests were completed at PRE, IP, and 60P during both experimental trials (visits 4 and 5). Each test battery took approximately 30 min to complete with scoring and testing administered in accordance with the ANAM test manual. All ANAM subtests provided throughput scores (TP) that were used for analyses, except for the Go/No-Go test and the Concussion Symptoms Inventory (CSI). Throughput (defined as LegacyThru in ANAM^™^ software), represents the rate of correct responses per minute, and is calculated using accuracy and speed variables, where speed is calculated by dividing 60,000 by the mean reaction time for all valid responses. Throughput is considered a measure of effectiveness or cognitive efficiency (24). For the Go/No-Go test, D-prime scores were used, which represents the most comprehensive measure of accuracy on go and no-go trials by assessing the ability to detect and respond quickly to appropriate stimuli and inhibit inappropriate responses (25, 26).

### Supplementation protocol

Subjects were assigned to consume either PeakATP^®^ or placebo (PLA) for a 14-day period prior to the completion of each experimental trial in a randomized, double-blind, cross-over fashion. Assignment of treatments were matched by sex across the two conditions. Subjects also ingested an acute dose of the assigned supplement upon arrival to the lab at the beginning of each experimental trial. PeakATP^®^ (400 mg adenosine 5′-triphosphate disodium, maltodextrin, silica-colloidal anhydrous, citric acid anhydrous sucralose & guar gum) and PLA (maltodextrin, silica-colloidal anhydrous, citric acid anhydrous, sucralose & guar gum) were obtained from TSI Group Ltd. (Missoula, MT, United States). Both PeakATP^®^ and PLA were provided in pre-portioned single serve stick packs in the form of a flavored powder that were similar in taste and appearance. Each participant was provided with a 14-day supply of their assigned formula following visit 3 and experimental trial 1 (visit 4). Subjects were instructed to mix their assigned formula in 250 mL of water and take 30 min before breakfast on an empty stomach. Subjects were required to keep a daily log detailing the date and time for which each dose was ingested, as well as any perceived side effects associated with the supplement. Subjects were required to return all empty packets before the beginning of each experimental trial. Any remaining supplement was counted and recorded. Supplement compliance was deemed as consuming at least 90% of each assigned supplement (missing <2 doses). During each experimental trial (visits 4 and 5), the supplement was taken immediately upon arrival to the lab 30 min prior to pre-testing. Supplements were coded, assigned, and administered in a double-blind fashion. A sealed envelope containing the identity of each coded supplement was provided by the supplement manufacturer prior to the beginning of the study. Blinding was maintained throughout data collection and analysis and was unblinded upon completion of the statistical analysis.

### Statistical analyses

Prior to any statistical analyses, data was assessed for normality using individual skewness and kurtosis scores, as well as the Shapiro Wilks test. All non-normally distributed data were transformed using a log10 transformation and rechecked for normality. If data remained non-normally distributed, non-transformed data was used for analysis. Data were then assessed for sphericity. If the assumption of sphericity was violated, a Greenhouse-Geiser correction was applied. A two-way treatment × time repeated measures ANOVA was then conducted to compare all dependent variables between treatments across time. When a significant interaction occurred, Fisher’s LSD pairwise comparisons were utilized to assess differences between treatment across time. Where there was no significant interaction between treatments, main effects were reported. Effects were further analyzed using partial eta-squared (η_p_^2^) and Hedges’ *g* (*g*) effect sizes. Partial eta-squared were evaluated in accordance with Cohen (27) at the following levels: small effect (0.01–0.058), medium effect (0.059–0.137), and large effect (>0.138). Hedges *g* were interpreted using thresholds of <0.2, 0.2 to <0.6, 0.6 to <1.2, 1.2 to <2.0, and 2.0–4.0, which correspond to trivial, small, moderate, large, and very large effect sizes respectively. Since estimates for *g* may show positive bias with small sample sizes, a correction was applied to provide a more accurate estimate of effect size (Eq 2) (28).


(2) $$g=\frac{{\bar{X}}_{2}-{\bar{X}}_{1}}{\sqrt{\frac{\left(n_{1}-1\right)S_{1}^{2}+\left(n_{2}-1\right)S_{2}^{2}\;}{\left(n_{1}-1\right)+\left(n_{2}-1\right)}}}\;x\;1-\frac{3}{4n-9},$$

Where n = number of observations for each time point/treatment and s = SD of the observations. For all statistical tests, data were considered statistically significant when the probability of error was 0.05 or less (*p* ≤ 0.05). All statistical analyses were completed using SPSS statistical software (v. 28.0.1.1).

## Results

### Subject compliance

All subjects were found to be in compliance with the supplementation protocol. Compliance for the PeakATP^®^ treatment was 98.6% and was 96.9% for the Placebo treatment.

### Reaction time

All data were normally distributed. Changes in Dynavision Mode A (Proactive) and Mode B (Reactive) variables during both experimental treatments are provided in Table 2. Interpretation of effect sizes are provided in Appendix 1 (see Table, Supplemental Digital Content 1, Effect sizes between time points within each treatment for reaction time, multiple object tracking, and mood assessment variables).

**Table 2:** Dynavision task assessments.

Dynavision task	Treatment	PRE	IP	60P	TRT × Time
Mode A hits	PeakATP	93.30 ± 8.35	91.95 ± 8.00	93.57 ± 9.31	0.006
	PLA	94.83 ± 8.24	91.80 ± 7.65^*^	91.03 ± 8.80*^§^	
Mode A AvgRT (sec)	PeakATP	0.647 ± 0.059	0.657 ± 0.059	0.646 ± 0.066	0.006
	PLA	0.638 ± 0.057	0.658 ± 0.057^*^	0.666 ± 0.069*^§^	
Mode B hits	PeakATP	77.88 ± 11.14	79.13 ± 11.92	79.03 ± 12.03	0.181
	PLA	78.55 ± 11.01	77.37 ± 11.00	78.68 ± 10.54	
Mode B AvgRT (sec)	PeakATP	0.663 ± 0.052	0.651 ± 0.049^*^	0.649 ± 0.050*	0.039
	PLA	0.656 ± 0.043	0.658 ± 0.045	0.656 ± 0.041	
Mode B misses^‡^	PeakATP	7.55 ± 4.21	9.05 ± 4.54	7.50 ± 4.99	0.612
	PLA	8.25 ± 4.44	9.85 ± 6.16	9.40 ± 5.01	

Data presented as mean ± standard deviation. PRE, pre-exercise; IP, immediately post-exercise; 60P, 60 min post-exercise; PLA, placebo; AvgRT, average reaction time; TRT × Time, *p*-value for treatment × time interaction effect; *, significantly different than PRE; ^‡^, significant main effect of treatment; ^§^, significantly different between treatments.

#### Mode A

##### Hits

**Figure 3:**
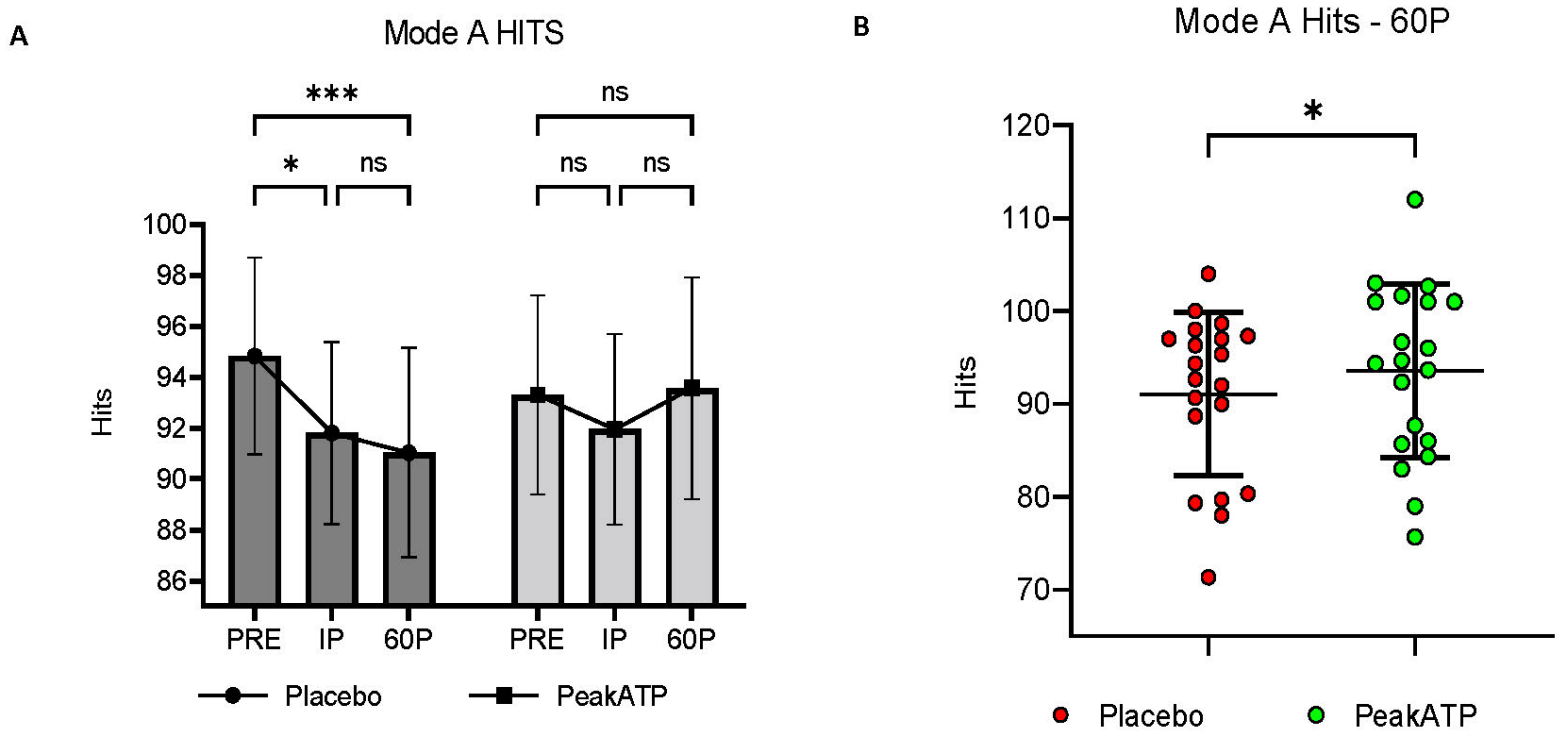
Mode A Hits. **(A)** Changes within treatments across time, **(B)** Between treatment differences at 60P. A significant treatment × time interaction was observed for Mode A hits (F_2,38_ = 5.85, *p* = 0.006, η_p_^2^ = 0.235). PRE, pre-exercise; IP, immediately post-exercise; 60P, 60 min post-exercise; ns, not significant; *** *p* = <0.001; **p = <0.01; * *p* = <0.05.

A significant treatment × time interaction was observed for Mode A hits (F_2,38_ = 5.85, *p* = 0.006, η_p_^2^ = 0.235). In placebo, the number of hits was significantly lower at IP (*p* = 0.019, *g* = −0.374) and 60P (*p* < 0.001, *g* = −0.437) compared to PRE, whereas the number of hits was maintained in PeakATP^®^ across all time points (*p*’s > 0.05) (Figure 3A). The number of hits was significantly greater in PeakATP^®^ at 60P when compared to placebo (*p* = 0.028, *g* = −0.274) (Figure 3B).

##### AvgRT

**Figure 4:**
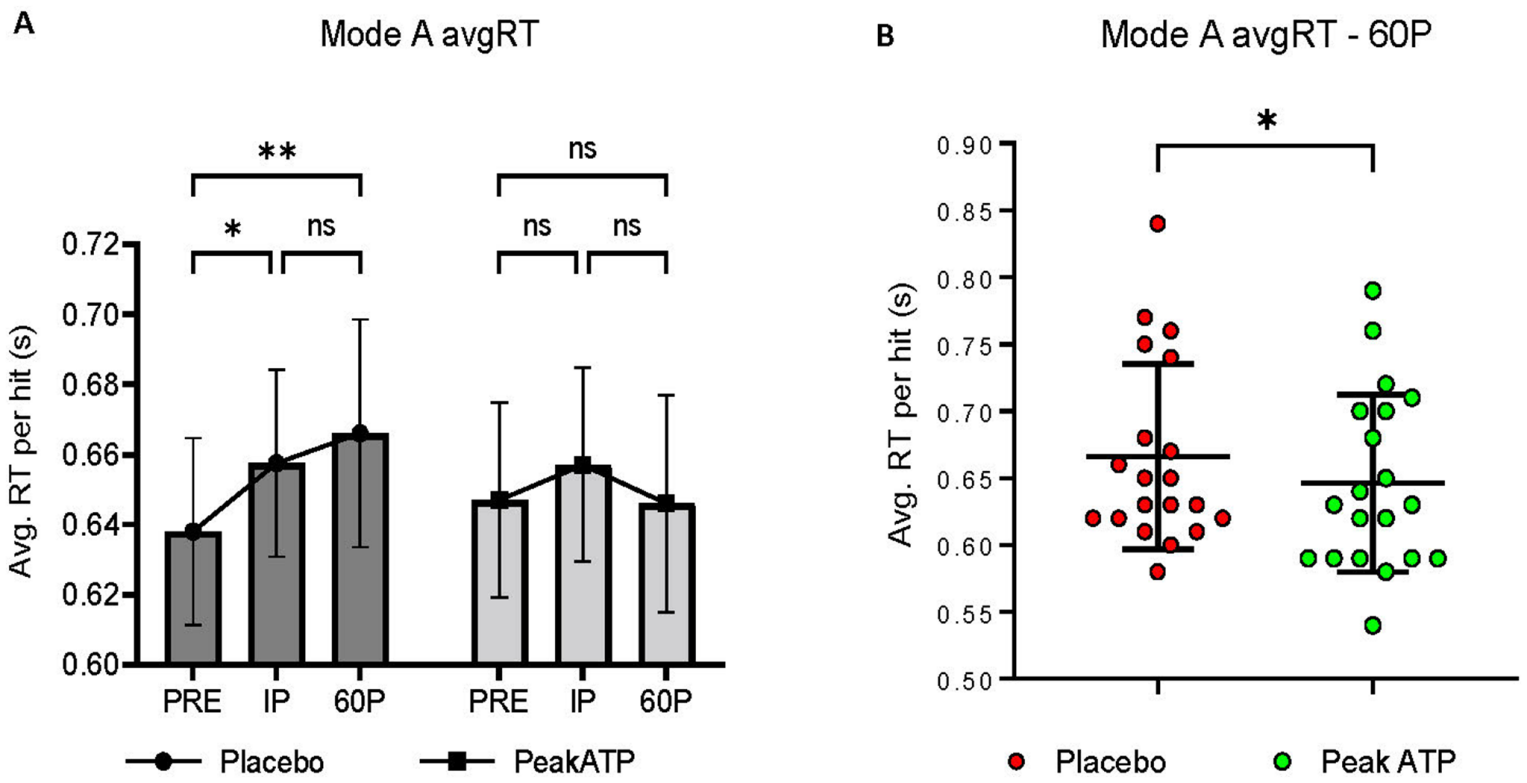
**(A)** Changes within treatments across time, **(B)** Between treatment differences at 60P. A significant treatment × time interaction was noted for Mode A average reaction time (F_2,38_ = 5.865, *p* = 0.006, η_p_^2^ = 0.236). AvgRT, average reaction time; PRE, pre-exercise; IP, immediately post-exercise; 60P, 60 min post-exercise; s, seconds; ns, not significant; * *p* = <0.05; ** *p* = <0.01.

A significant treatment × time interaction was noted for Mode A AvgRT (F_2,38_ = 5.865, *p* = 0.006, η_p_^2^ = 0.236). In placebo, AvgRT was significantly slower at IP (*p* = 0.027, *g* = 0.336) and 60P (*p* = 0.002, *g* = 0.433) compared to PRE, whereas AvgRT was maintained in PeakATP^®^ (Figure 4A). No differences were noted between treatments at PRE or IP (*p*’s > 0.05). AvgRT was significantly faster in PeakATP^®^ at 60P when compared to placebo (*p* = 0.015, *g* = 0.289) (Figure 4B).

#### Mode B

##### Hits

No significant treatment × time interaction (F_2,38_ = 1.786, *p* = 0.181, η_p_^2^ = 0.086), main effect for treatment (F_1,38_ = 0.261, *p* = 0.615, η_p_^2^ = 0.014) or main effect for time (F_2,38_ = 0.404, *p* = 0.671, η_p_^2^ = 0.021) was noted for number of hits in Mode B.

##### AvgRT

**Figure 5:**
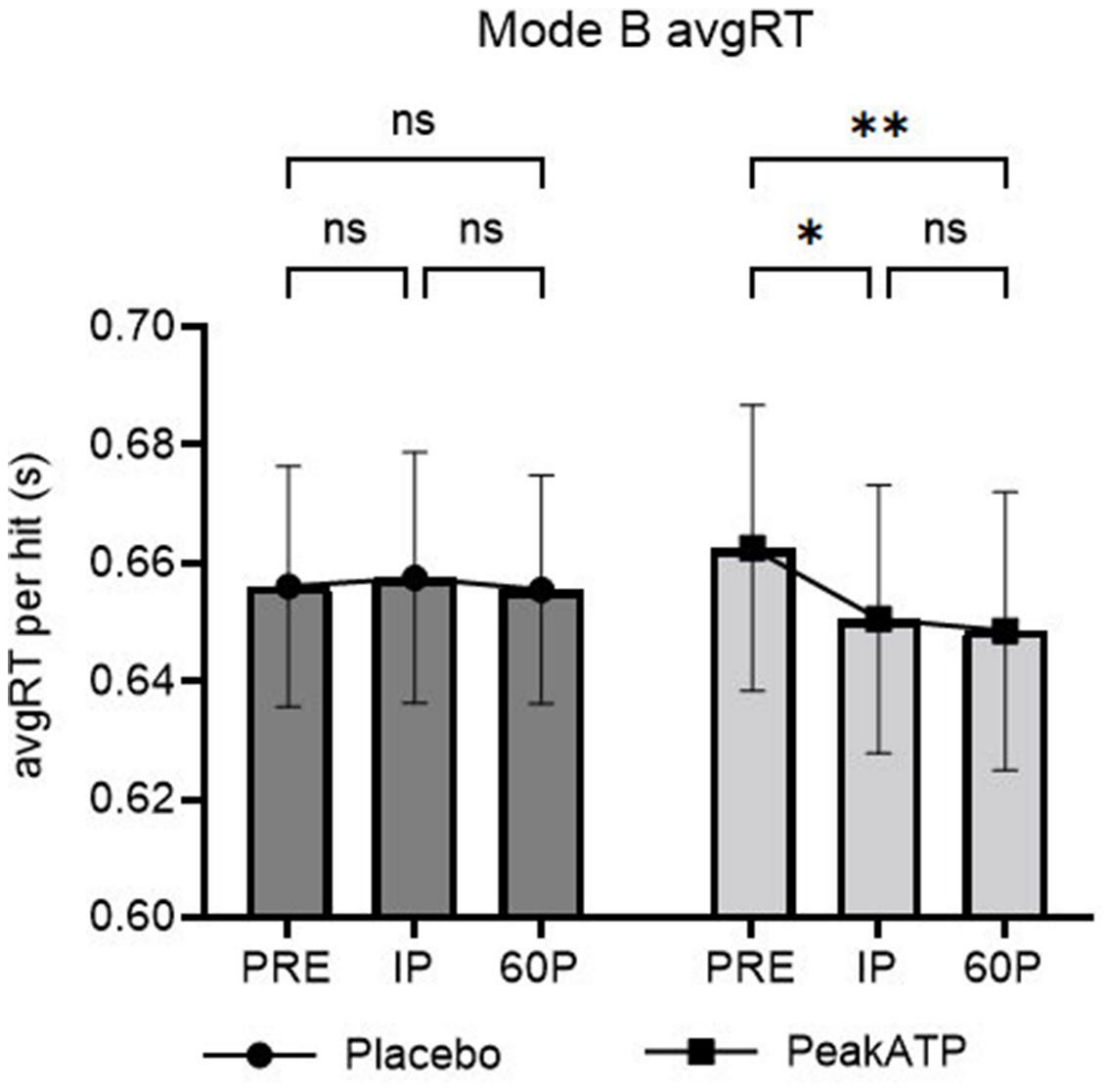
Mode B changes within treatments across time for average reaction time. A significant treatment × time interaction was noted for Mode B average reaction time (F_2,38_ = 4.014, *p* = 0.039, η_p_^2^ = 0.174). AvgRT, average reaction time; PRE, pre-exercise; IP, immediately post-exercise; 60P, 60 min post-exercise; s, seconds; ns, not significant; **p = <0.01; * *p* = <0.05.

A significant treatment × time interaction was noted for AvgRT (F_2,38_ = 4.014, *p* = 0.039, η_p_^2^ = 0.174). In PeakATP^®^, AvgRT was significantly faster at IP (*p* = 0.015, *g* = −0.235) and 60P (*p* = 0.001, *g* = −0.269) compared to PRE (Figure 5). No differences were noted between timepoints for placebo (*p*’s > 0.05).

##### Misses

No significant treatment × time interaction was noted for number of misses in Mode B (F_2,38_ = 0.497, *p* = 0.536, η_p_^2^ = 0.026). However, significant main effects for time (F_2,38_ = 3.302, *p* = 0.048, η_p_^2^ = 0.148) and treatment (F_1,38_ = 9.921, *p* = 0.005, η_p_^2^ = 0.343) were noted. Regardless of treatment, the number of misses increased at IP compared to PRE (*p* = 0.018, *g* = 0.100). However, the number of misses was significantly lower in PeakATP^®^ overall when compared to placebo (F_1,38_ = 9.921, *p* = 0.005, η_p_^2^ = 0.343).

### Multiple object tracking

All data were normally distributed. Changes in MOT speed during both experimental treatments are provided in Table 3. Interpretation of effect sizes are provided in Appendix 1 (see Table, Supplemental Digital Content 1, Effect sizes between time points within each treatment for reaction time, multiple object tracking, and mood assessment variables). No significant treatment × time interaction (F_2,38_ = 0.161, *p* = 0.852, η_p_^2^ = 0.008), or main effect for treatment (F_1,38_ = 1.364, *p* = 0.257, η_p_^2^ = 0.067) was observed. However, a significant main effect for time was noted (F_2,38_ = 3.362, *p* = 0.045, η_p_^2^ = 0.150) with MOT speed being greater at 60P compared to PRE, regardless of treatment (*p* = 0.038 *g* = 0.100).

**Table 3:** Neurotracker multiple object tracking speed.

Neurotracker task	Treatment	PRE	IP	60P
MOT Speed (cm/s)*	PeakATP	1.245 ± 0.636	1.290 ± 0.354	1.389 ± 0.440
	PLA	1.236 ± 0.459	1.223 ± 0.402	1.330 ± 0.461

Data presented as means ± standard deviation. MOT, multiple object tracking; PRE, pre-exercise; IP, immediately post-exercise; 60P, 60 min post-exercise; PLA, placebo; *, 60P significantly different than PRE.

### Mood states (POMS)

Vigor and total mood disturbance were normally distributed. All other POMS variables were non-normal. Changes in Profile of Mood States during the experimental trials are reported in Table 4. Interpretation of effect sizes are provided in Appendix 1 (see Table, Supplemental Digital Content 1, Effect sizes between time points within each treatment for reaction time, multiple object tracking, and mood assessment variables). No significant treatment × time interaction, or main effect for treatment was noted for any mood state variable. Significant main effects for time were noted for tension (F_2,38_ = 5.534, *p* = 0.014, η_p_^2^ = 0.226) and fatigue (F_2,38_ = 6.145, *p* = 0.017, η_p_^2^ = 0.244). Regardless of treatment, tension was significantly lower at 60P compared to PRE (*p* < 0.001, *g* = −0.278) and at 60P compared to IP (*p* = 0.018, *g* = −0.182), while fatigue was significantly lower at 60P compared to IP (*p* = 0.002, *g* = −0.556). No other significant time effects were noted for mood state variables (*p’s* > 0.05).

**Table 4:** Changes in mood states as assessed via the profile of mood states questionnaire.

Mood state	Treatment	PRE	IP	60P	TRT × Time
Tension*^†^	PeakATP	38.90 ± 7.64	38.65 ± 9.29	36.90 ± 7.67	0.834
	PLA	39.25 ± 6.45	38.50 ± 9.12	37.20 ± 7.36	
Depression	PeakATP	38.00 ± 2.18	38.10 ± 2.88	37.70 ± 2.25	0.497
	PLA	38.25 ± 2.63	37.90 ± 2.79	37.55 ± 1.64	
Anger	PeakATP	39.55 ± 5.86	40.15 ± 8.43	39.65 ± 6.40	0.230
	PLA	39.20 ± 5.88	39.05 ± 7.19	39.50 ± 6.77	
Vigor	PeakATP	47.75 ± 10.77	47.40 ± 11.86	46.25 ± 13.25	0.776
	PLA	46.45 ± 11.69	47.50 ± 13.66	46.60 ± 13.18	
Fatigue^†^	PeakATP	38.35 ± 5.96	41.25 ± 8.18	38.40 ± 6.63	0.234
	PLA	39.50 ± 7.48	44.30 ± 10.53	38.20 ± 5.38	
Confusion	PeakATP	36.55 ± 5.01	35.85 ± 6.22	35.20 ± 6.62	0.623
	PLA	35.10 ± 5.12	35.45 ± 7.10	34.75 ± 6.32	
TMD	PeakATP	243.60 ± 21.22	246.60 ± 31.63	241.60 ± 27.22	0.895
	PLA	244.85 ± 28.02	247.70 ± 33.00	240.60 ± 22.61	

Data are presented as means ± standard deviation. PRE, pre-exercise; IP, immediately post-exercise; 60P, 60 min post-exercise; PLA, placebo; TRT × Time, *p*-value for treatment × time interaction effect; TMD, total mood disturbances, main Effect for time collapsed across treatments: *, 60P Significantly different than PRE; ^†^, 60P significantly different than IP.

### Automated neuropsychological assessment metrics

All ANAM data were normally distributed. Changes in ANAM core battery variables during the exercise trials are provided in Table 5. Interpretation of effect sizes are provided in Appendix 2 (see Table, Supplemental Digital Content 2, Effect sizes between time points within each treatment for ANAM cognitive assessment variables).

**Table 5:** Changes in automated neuropsychological assessment metrics (ANAM).

ANAM task	Treatment	PRE	IP	60P	TRT × Time
SRT (TP)	PeakATP	217.98 ± 18.86	215.02 ± 22.25	215.68 ± 19.34	0.493
	PLA	215.77 ± 22.74	204.99 ± 37.39	211.84 ± 24.25	
CS (TP)	PeakATP	63.62 ± 8.91	61.50 ± 10.14	62.46 ± 11.43	0.492
	PLA	62.34 ± 11.32	57.91 ± 11.69	62.35 ± 9.51	
PRT (TP)*	PeakATP	102.46 ± 10.40	103.94 ± 11.20	108.28 ± 17.18	0.994
	PLA	98.90 ± 13.34	100.17 ± 16.74	104.95 ± 11.74	
MATH (TP)*^†^	PeakATP	28.61 ± ± 8.16	28.75 ± 10.00	30.11 ± 8.99	0.358
	PLA	27.62 ± 10.08	25.88 ± 8.52	30.84 ± 8.43	
M2S (TP)^^†^	PeakATP	43.47 ± 11.69	39.90 ± 9.21	43.28 ± 14.23	0.934
	PLA	41.67 ± 13.38	37.71 ± 12.19	41.62 ± 11.23	
CSD (TP)^	PeakATP	55.97 ± 14.10	50.59 ± 15.38	53.32 ± 18.03	0.334
	PLA	56.70 ± 14.26	44.99 ± 13.42	53.09 ± 16.62	
SRT2 (TP)	PeakATP	207.70 ± 20.25	206.81 ± 25.67	203.56 ± 20.25	0.311
	PLA	203.85 ± 28.63	207.22 ± 25.84	208.07 ± 21.87	
GNG (D-Prime)^*	PeakATP	4.44 ± 1.73	3.82 ± 1.56	3.57 ± 1.41	0.877
	PLA	4.45 ± 1.72	3.66 ± 1.44	3.66 ± 1.39	
CSI_%_	PeakATP	14.5 ± 19.8	22.4 ± 27.5	14.0 ± 21.0	0.347
	PLA	18.9 ± 20.6	23.2 ± 17.9	11.8 ± 14.0	
CSI_SUM_	PeakATP	2.9 ± 4.5	5.1 ± 7.2	3.5 ± 6.2	0.235
	PLA	4.8 ± 7.1	5.6 ± 6.3	2.7 ± 4.7	

Data are presented as means ± standard deviation. TP, Throughput: LegacyThru (the rate of correct responses per minute); PRE, pre-exercise; IP, immediately post-exercise; 60P, 60 min post-exercise; PLA, placebo; TRT × Time, *p*-*value for* treatment × time interaction effect; SRT, simple reaction time; CS, code substitution; PRT, procedural reaction time; MATH, mathematical processing; M2S, matching to sample; CSD, code substitution delayed; SRT2, simple reaction time repeat; CSI_%_, concussion symptom inventory—percentage of endorsed symptoms; CSI_SUM_, concussion symptom inventory—sum of severity ratings; main effect for time collapsed across treatments: ^, IP Significantly different than PRE; *, 60P Significantly different than PRE; ^†^, 60P Significantly different than IP.

#### Concussion symptoms inventory

No significant treatment × time interactions, main effects for treatment or main effects for time were noted for the percentage of endorsed symptoms or the sum of severity ratings for CSI symptoms (*p*’s > 0.05).

#### ANAM cognitive tasks

No significant treatment × time interactions or main effects for treatment were noted for throughput scores in any of the cognitive tasks (*p*’s > 0.05). Main effects for time were noted for PRT (F_2,36_ = 3.388, *p* = 0.045, η_p_^2^ = 0.158), MATH (F_2,36_ = 7.758, *p* = 0.002, η_p_^2^ = 0.301), M2S (F_2,36_ = 3.671, *p* = 0.035, η_p_^2^ = 0.169), and CSD (F_2,36_ = 4.610, *p* = 0.019, η_p_^2^ = 0.204). Regardless of treatment, TP was significantly lower at IP compared to PRE in the M2S (*p* = 0.016, *g* = −0.281) and CSD (*p* = 0.001, *g* = −0.611) tasks, significantly higher at 60P compared to PRE in the PRT (*p* = 0.007, *g* = 0.453) and MATH (*p* = 0.013, *g* = 0.263) tasks, and significantly higher at 60P compared to IP in the MATH (*p* = 0.001, *g* = 0.348), and M2S (*p* = 0.015, *g* = 0.298) tasks. No other significant main effects for time were noted for ANAM cognitive tasks.

#### Go/No-Go D-prime

No significant time × treatment interaction (F_2,36_ = 0.131, *p* = 0.877, η_p_^2^ = 0.007) or main effect for treatment (F_1,36_ = 0.010, *p* = 0.923, η_p_^2^ = 0.001) were noted for D-Prime in the GNG task. However, a significant main effect for time was noted (F_2,36_ = 5.832, *p* = 0.013, η_p_^2^ = 0.245). Regardless of treatment, D-Prime was significantly lower at IP (*p* = 0.007, *g* = −0.450) and 60P (*p* = 0.021, *g* = −0.533) when compared to PRE.

## Discussion

This study sought to examine the effects of daily supplementation with 400 mg of PeakATP^®^ on RT, cognitive function, MOT, and mood following a 3MT. In the current trial, significant post-exercise deficits were noted for M2S and CSD at IP, for Go/No-Go D-Prime at IP and 60P, and for number of misses in the Dynavision Mode B assessment at IP compared to PRE. Further, declines in number of hits and AvgRT were noted at IP and 60P for the Dynavision Mode A task following the placebo treatment. Together, these results indicate that the 3MT elicited immediate and transient post-exercise deficits (up to 60-min) for several cognitive tasks and was therefore an appropriate stimulus for causing acute cognitive dysfunction. Findings relating to the primary outcomes indicated that 14-days supplementation with PeakATP^®^ in combination with an acute dose 30 min prior to pre-exercise testing, significantly attenuated the decline in the number of hits and AvgRT per hit in the Dynavision Mode A assessment when compared to placebo. AvgRT per hit in the Dynavision Mode B assessment was also significantly improved in PeakATP^®^, while the number of misses was significantly lower overall for PeakATP^®^ when compared to placebo. These findings indicate that PeakATP^®^ may positively influence RT following an acute bout of HISE. Nevertheless, ANAM, MOT and mood variables were not significantly different between PeakATP^®^ and placebo following the exercise bout.

Reaction time, which is an extension of perception and anticipation, bridges motor action to the presentation of a specific stimulus (29), and thus represents a practical and measurable component of many cognitive assessments that can be utilized along side lab based exercise protocols. The benefits of PeakATP^®^ on RT in the current study are in contrast with the findings of a similar study by Purpura et al. (13) who reported that an identical supplemental regimen of PeakATP^®^ did not impact a visuomotor RT assessment on the Dynavision immediately following a 10 × 6 s repeated Wingate protocol. However, the RT task utilized by Purpura et al. was less complex, and therefore potentially less sensitive to change compared to the Mode A and Mode B tasks utilized in the present study. Consistent with this, the comparatively simpler ANAM SRT subtask utilized in the present investigation was not influenced by the exercise bout. Similar results were previously observed by Bue-Estes and colleagues who reported that ANAM SRT was not effected by short-term maximal treadmill running (30). Differences in results may therefore be related to the complexity of RT assessments or the exercise intervention itself. Notably, the present study expanded on the Purpura investigation (13) by including additional post-exercise assessments at 60P, which establishes that post-exercise deficits in RT may be observed for up to 1-h after intense exercise, and are subject to perturbation by ATP supplementation. The persistence of cognitive dysfunction for up to 60 min following a single bout of HISE is noteworthy for coaches, as this may have implications for action anticipation and decision making ability among athletes, particularly towards the latter part of a competitive game/match. While our findings provide evidence for immediate and transient deficits in cognitive function, whether these deficits are compounded following repeated bouts is unknown. Future studies may consider the impact of repeated bouts of exhaustive HISE on cognitive performance in conjunction with several post-exercise assessments to fully capture the impact of both exercise and supplement interventions on visuomotor RT assessments.

Several studies have demonstrated that computerized cognitive assessments are sensitive to changes in cognitive function following exercise (6, 28, 30, 31). Similar to the present study, Mekari et al. (6) reported significantly slower reaction time and accuracy scores on a computer based Modified Stroop executive function task during high intensity cycling exercise when compared to the non-executive task. Regardless of task type (executive vs. non-executive), higher exercise intensity resulted in slower reaction times and decreased accuracy, as well as reduced cerebral oxygenation. Bue-Estes et al. (30) reported no deficits in several ANAM subtasks (SRT, CSL, CSD and continual processing) immediately following an acute short-term maximal bout of treadmill running. However, deficits in MATH subtask were observed immediately post-exercise, along with a subsequent improvement in MATH and M2S tasks following 30 min of recovery compared to immediately post-exercise values. In contrast, the present investigation did not observe a significant deficit in MATH performance immediately post-exercise, while the improvement in MATH following a period of recovery was comparable between investigations. Both Wells et al. (28) and Varanoske et al. (31), reported decreased ANAM subtask performance and a greater number and severity of CSI symptoms towards the end of a 24-h simulated military operation that included military type tasks and activities in conjunction with caloric restriction and sleep restriction.

To this extent, the differences in study designs, exercise intensity, modality, duration, populations assessed, and timing or type of cognitive assessments may have impacted the extent to which cognitive dysfunction via ANAM or computer-based assessments could be elicited, as these factors are considered to be moderators of the cognition and acute exercise paradigm (32). It is also possible that computerized cognitive assessments such as the ANAM, may not evoke a significant ergogenic opportunity due to the lack of musculoskeletal recruitment or vestibular challenge required to perform them (33). Substantial evidence exists suggesting that the vestibular system impacts cognitive processing and attention, with computerized assessments unable to stimulate an individual’s peripheral vestibular system (33). Moreover, previous literature has indicated that when assessing cognitive performance in healthy volunteers, it is of the utmost importance to account for differences in circadian preference, as time-of-day may impact performance (34, 35). Consistent with this, all subjects in the present study were tested at the same time of day for both PeakATP^®^ and placebo treatments.

PeakATP^®^ did not have a significant impact on MOT scores, although a significant main effect indicating improved performance regardless of treatment was observed. A previous investigation by Klotzbier et al. (36) reported similar improvements in MOT scores in young adults prior to and during acute intermittent treadmill exercise at three time points. This suggests that repeated MOT testing may also result in a learning or training effect like that reported in the Dynavision reaction time tasks, or perhaps was the result of an exercise-induced improvement that may persist post-exercise. Additional research, akin to the investigation performed by Wells and Johnson (19) for the Dynavision Mode A RT assessment appears necessary to determine the appropriateness of the MOT device as an acute cognitive assessment, rather than as a training tool. PeakATP^®^ also did not have a significant impact on indices of mood. However, tension was found to be significantly reduced at 60P compared to PRE and IP. Previous literature has reported that exercise is an effective tool for mitigating tension with acute bouts of exercise (37), which is in line with the current findings. Additionally, fatigue was reported to be significantly reduced at 60P compared to IP, with no differences from at IP or 60P compared to PRE. This suggests that there was an increase in fatigue from PRE to IP that did not reach significance and is supported by the moderate effect size for an increase in fatigue with the placebo treatment (*g* = 0.878).

Previous research examining ATP supplementation and exercise suggests that ATP may impact fatigue (12), energy expenditure (10) and athletic performance (10–12, 38–40), although we have previously shown that ATP supplementation does not influence 3MT performance (18). The present investigation is the first to examine the impact of ATP on cognitive function, although the intersection of the proposed mechanisms through which ATP may impact cognitive function (alterations in regional cerebral oxygenation, blood flow, or ATP synthesis), were not examined. Consequently, the mechanism underpinning the observed improvements in visuomotor reaction time following PeakATP^®^ supplementation is unclear. Future investigations should aim to expand on these findings, by assessing potential mechanisms of ATP, in addition to performance metrics of cognition within several different populations.

There are several limitations in the current study. Firstly, we utilized a heterogenous sample of men and women with varying degrees of training experience and activity levels. The difference in training experience could have influenced the degree to which cognitive dysfunction was elicited, as those with greater training experience may be more equipped to recognize and exert maximal effort during exercise interventions compared to their lesser trained counterparts (41). However, by utilizing a counterbalanced and randomized trial, effects associated with a learning curve of the exercise intervention should have been mitigated. Secondly, it is possible that participants who were consumers of caffeine may have experienced symptoms of acute caffeine withdrawal during the 24-h period prior to each trial. In consideration of the half-life of caffeine, we elected to utilize a 24-h period of caffeine abstinence since even moderate doses of caffeine can remain in the plasma for up to 12 h (42). This should have mitigated any potential beneficial effects of caffeine on cognitive outcomes; however, negative effects associated with withdrawal are possible. Nevertheless, the period of abstinence was consistent within subjects between trials, so we expect any effects of potential caffeine withdrawal were also consistent between trials. Thirdly, we did not monitor hydration status or caloric intake prior to each trial. The influence of hydration on cognitive performance in the literature is divergent and cognitive deficits are generally only observed following >2% dehydration in response to water restriction in conjunction with either heat exposure or prolonged exercise (43). In the current study, we did not induce a state of dehydration prior to exercise and participants were provided with 8 oz. water 30-min prior to pre-testing during each trial. Therefore, we do not expect that the hydration status of participants had a significant influence on cognitive outcomes. The influence of differences in caloric intake on cognitive outcomes during the hours preceding each trial also cannot be ruled out. However, participants were required to be 2-h fasted prior to arrival at the lab, and we observed no significant baseline differences in fatigue or vigor between trials. Further, we previously reported no differences in physical performance during the 3MT between trials among the same individuals (18) and therefore expect that any differences relating to caloric intake would have been evident in those findings. Finally, methodologies to examine the potential mechanisms associated with ergogenic effects of ATP disodium were not implemented, therefore, the present study is unable to reconcile with the mechanistic findings of previous studies.

In conclusion, the present study is the first to examine whether daily supplementation with 400 mg of PeakATP^®^ for a period of 14 days in combination with an acute dose prior to HISE differentially impacts RT, cognitive function, MOT, and mood when compared to placebo. Supplementation with oral PeakATP^®^ attenuated the declines in proactive visuomotor RT, improved reactive visuomotor RT, and decreased the number of misses during the reactive visuomotor assessments. While the results of the present study are novel and additional research is needed, active individuals may wish to consider utilizing supplemental PeakATP^®^ as a potential mitigator of HISE induced cognitive dysfunction, specifically with regards to deficits in reaction time. However, the potential mechanisms driving these ergogenic effects remain unknown. Future research should consider implementing methodologies to examine changes in potential mechanistic variables in addition to supplementary strategies to measure cognitive function following exercise.

## Data availability statement

The raw data supporting the conclusions of this article will be made available by the authors, without undue reservation.

## Ethics statement

The studies involving humans were approved by University of Central Florida Institutional Review Board. The studies were conducted in accordance with the local legislation and institutional requirements. Written informed consent for participation in this study was provided by the participants’ legal guardians/next of kin.

## Author contributions

AW designed the study. JM and TD collected the data under the supervision of AW. AW and JM analyzed the data and wrote the initial manuscript draft. All authors contributed to the article and approved the submitted version.

## Funding

This study was funded by TSI Group Ltd. The sponsors had no role in collecting the data, analyzing the data, interpreting the results, or preparing the manuscript.

## Conflict of interest

The authors declare that the research was conducted in the absence of any commercial or financial relationships that could be construed as a potential conflict of interest.

## Publisher’s note

All claims expressed in this article are solely those of the authors and do not necessarily represent those of their affiliated organizations, or those of the publisher, the editors and the reviewers. Any product that may be evaluated in this article, or claim that may be made by its manufacturer, is not guaranteed or endorsed by the publisher.
