# Supplementary material for: Evaluating the effects of PeakATP® supplementation on visuomotor reaction time and cognitive function following high-intensity sprint exercise
Source: Front Nutr. 2023 Aug 4;10:1237678. doi: 10.3389/fnut.2023.1237678 (PMC10436484; doi:10.3389/fnut.2023.1237678)
Supplement: Supplementary file 1 [file Data_Sheet_1.PDF]

Supplementary Digital Content 1. Appendix 1: Effect sizes between time points within each treatment for reaction time, multiple object tracking, and mood assessment variables.

**Variable**	**Treatment**	PRE vs IP	PRE vs 60P	IP vs 60P
**Mode A Hits**	PeakATP	-0.162 (trivial effect)	0.030 (trivial effect)	0.183 (trivial effect)
	PLA	-0.374 (small effect)	-0.437 (small effect)	-0.091(trivial effect)
**Mode A avgRT (sec)**	PeakATP	0.166 (trivial effect)	-0.016 (trivial effect)	-0.172 (trivial effect)
	PLA	0.336 (small effect)	0.433(small effect)	0.131(trivial effect)
**Mode B Hits**	PeakATP	0.106 (trivial effect)	0.097 (trivial effect)	-0.008 (trivial effect)
	PLA	-0.105 (trivial effect)	0.012 (trivial effect)	0.120 (trivial effect)
**Mode B avgRT (sec)**	PeakATP	-0.235 (small effect)	-0.269 (small effect)	-0.040 (trivial effect)
	PLA	0.033 (trivial effect)	-0.012 (trivial effect)	-0.045 (trivial effect)
**Mode B misses**	PeakATP	0.336 (small effect)	-0.011(trivial effect)	-0.319 (small effect)
	PLA	0.292 (small effect)	0.238 (small effect)	-0.079 (trivial effect)
**MOT Speed**	PeakATP	0.122 (trivial effect)	0.349 (small effect)	0.243 (small effect)
	PLA	-0.031 (trivial effect)	0.199 (trivial effect)	0.243 (small effect)
**Tension**	PeakATP	-0.029 (trivial effect)	-0.256 (small effect)	-0.201 (small effect)
	PLA	-0.093 (trivial effect)	-0.290 (small effect)	-0.154 (trivial effect)
**Depression**	PeakATP	0.038 (trivial effect)	-0.133 (trivial effect)	-0.152 (trivial effect)
	PLA	-0.126 (trivial effect)	-0.313 (small effect)	-0.150 (trivial effect)
**Anger**	PeakATP	0.081 (trivial effect)	0.016 (trivial effect)	-0.066 (trivial effect)
	PLA	-0.022 (trivial effect)	0.046 (trivial effect)	0.063 (trivial effect)
**Vigor**	PeakATP	-0.030 (trivial effect)	-0.122 (trivial effect)	-0.090 (trivial effect)
	PLA	0.126 (trivial effect)	0.012 (trivial effect)	-0.108 (trivial effect)
**Fatigue**	PeakATP	0.397 (small effect)	0.008 (trivial effect)	-0.375 (small effect)
	PLA	0.515 (moderate effect)	-0.196 (trivial effect)	-0.715 (moderate effect)
**Confusion**	PeakATP	-0.122 (trivial effect)	-0.225 (small effect)	-0.099 (trivial effect)
	PLA	0.055 (trivial effect)	-0.060 (trivial effect)	-0.102 (trivial effect)
**TMD**	PeakATP	0.109 (trivial effect)	-0.080 (trivial effect)	-0.166 (trivial effect)
	PLA	0.091 (trivial effect)	-0.164 (trivial effect)	-0.246 (small effect)

Data are presented as Hedge's *g* corrected for small sample size. Abbreviations: PRE= pre-exercise; IP= immediately post-exercise; 60P= 60 minutes post-exercise; PLA= placebo; avgRT= average reaction time; sec= seconds; TMD= total mood disturbances.
