# Supplementary material for: Evaluating the effects of PeakATP® supplementation on visuomotor reaction time and cognitive function following high-intensity sprint exercise
Source: Front Nutr. 2023 Aug 4;10:1237678. doi: 10.3389/fnut.2023.1237678 (PMC10436484; doi:10.3389/fnut.2023.1237678)
Supplement: Supplementary file 2 [file Data_Sheet_2.PDF]

Supplementary Digital Content 2. Appendix 2: Effect sizes between time points within each treatment for ANAM cognitive assessment variables.

**Variable**	**Treatment**	PRE vs IP	PRE vs 60P	IP vs 60P
**SRT (TP)**	PeakATP	-0.121 (trivial effect)	-0.110 (trivial effect)	0.019 (trivial effect)
	PLA	-0.362 (small effect)	-0.189 (trivial effect)	0.213 (small effect)
**CS (TP)**	PeakATP	-0.233 (small effect)	-0.146 (trivial effect)	0.065 (trivial effect)
	PLA	-0.340 (small effect)	0.045 (trivial effect)	0.409 (small effect)
**PRT (TP)**	PeakATP	0.197 (trivial effect)	0.422 (small effect)	0.266 (small effect)
	PLA	0.076 (trivial effect)	0.471 (small effect)	0.325 (small effect)
**MATH (TP)**	PeakATP	-0.011 (trivial effect)	0.148 (trivial effect)	0.143 (trivial effect)
	PLA	-0.169 (trivial effect)	0.361 (small effect)	0.574 (small effect)
**M2S (TP)**	PeakATP	-0.334 (small effect)	-0.032 (trivial effect)	0.258 (small effect)
	PLA	-0.234 (small effect)	0.065 (trivial effect)	0.327 (small effect)
**CSD (TP)**	PeakATP	-0.438 (small effect)	-0.254 (small effect)	0.132 (trivial effect)
	PLA	-0.787 (moderate effect)	-0.189 (trivial effect)	0.526 (small effect)
**SRT2 (TP)**	PeakATP	-0.081 (trivial effect)	-0.212 (small effect)	-0.124 (trivial effect)
	PLA	0.092 (trivial effect)	0.132 (trivial effect)	0.035 (trivial effect)
**GNG (D-Prime)**	PeakATP	-0.384 (small effect)	-0.537 (small effect)	-0.146 (trivial effect)
	PLA	-0.501 (small effect)	-0.507 (small effect)	0.001 (trivial effect)
**$CSI_{\%}$**	PeakATP	0.330 (small effect)	-0.020 (trivial effect)	-0.340 (small effect)
	PLA	0.252 (small effect)	-0.367 (small effect)	-0.696 (moderate effect)
**CSI_{SUM}**	PeakATP	0.357 (small effect)	0.092 (trivial effect)	-0.245 (small effect)
	PLA	0.130 (trivial effect)	-0.331 (small effect)	-0.510 (small effect)

Data are presented as Hedge's *g* corrected for small sample size. Abbreviations: PRE= pre-exercise; IP= immediately post-exercise; 60P= 60 minutes post-exercise; PLA= placebo; SRT= simple reaction time, CS= code substitution, PRT= procedural reaction time, MATH=mathematical processing, M2S= matching to sample, CSD= code substitution delayed, SRT2= simple reaction time repeat, $CSI_{\%}$= Concussion symptom inventory—percentage of endorsed symptoms; CSI_{SUM}= Concussion symptom inventory—sum of severity ratings; TP= Throughput: LegacyThru (the rate of correct responses per minute).
